# ‘Sensory and Motor Neuroscience’: Impacts of Thirteen Highly Cited Articles Published in This Section of *Brain Sciences* in 2024

**DOI:** 10.3390/brainsci16030292

**Published:** 2026-03-06

**Authors:** Bernadette Murphy

**Affiliations:** Faculty of Health Sciences, Ontario Tech University, Oshawa, ON L1G 0C5, Canada; bernadette.murphy@ontariotechu.ca

The most highly cited papers in this section span the breadth of sensorimotor neuroscience, with four studies related to neurodegeneration being highly cited. Topics of these papers included the role of nanoplastics and neurodegeneration in ALS, the Gold Coast criteria in ALS diagnosis, a narrative review of hypomimia (loss of spontaneous facial expressions) in Parkinson’s, and a revisiting of the why and how of animal models in Parkinson’s Disease. The growing importance of virtual reality (VR) to study sensorimotor function was evident in two highly cited reviews: one on the sense of agency and skills learning in a VR environment and the other reviewing the impact of VR on brain health. Topics related to rehabilitation were also highly ranked, including the importance of bodily awareness representation and interoception in neurorehabilitation, and hearing rehabilitation for single-sided deafness, involving signal devices, bone conduction devices and cochlear implants. Another highly cited paper compares the relationships between sensory processing and executive function in children with combined ASD and ADHD to typically developing and single-disorder participants. Three papers related to neuromusculoskeletal conditions were also highly cited: one was a clinical review comparing the similarities between neuralgic amyotrophy and hourglass nerve constriction/nerve torsion; another discussed the mechanisms behind sex differences in temporomandibular disorders and their comorbidity with migraine. Another migraine paper discussed the real-world application of calcitonin gene-related peptide monoclonal antibodies in the treatment of episodic and chronic migraine. Finally, a scoping review which sought to understand the effect of listening to, playing, and singing music using fNIRs rounded out the highly cited papers of 2024.

All articles published in our journal are available in an open access format, granting you unrestricted access to the full text for free. We invite you to explore our most highly cited papers of 2024, listed below.

